# Effect of P2X7 Receptor Knockout on AQP-5 Expression of Type I Alveolar Epithelial Cells

**DOI:** 10.1371/journal.pone.0100282

**Published:** 2014-06-18

**Authors:** Georg Ebeling, Robert Bläsche, Falk Hofmann, Antje Augstein, Michael Kasper, Kathrin Barth

**Affiliations:** 1 Institute of Anatomy, Medical Faculty Carl Custav Carus, Technische Universität Dresden, Dresden, Germany; 2 Department of Internal Medicine and Cardiology, Technische Universität Dresden, Dresden, Germany; Helmholtz Zentrum München/Ludwig-Maximilians-University Munich, Germany

## Abstract

P2X7 receptors, ATP-gated cation channels, are specifically expressed in alveolar epithelial cells. The pathophysiological function of this lung cell type, except a recently reported putative involvement in surfactant secretion, is unknown. In addition, P2X7 receptor-deficient mice show reduced inflammation and lung fibrosis after exposure with bleomycin. To elucidate the role of the P2X7 receptor in alveolar epithelial type I cells we characterized the pulmonary phenotype of P2X7 receptor knockout mice by using immunohistochemistry, western blot analysis and real-time RT PCR. No pathomorphological signs of fibrosis were found. Results revealed, however, a remarkable loss of aquaporin-5 protein and mRNA in young knockout animals. Additional *in vitro* experiments with bleomycin treated precision cut lung slices showed a greater sensitivity of the P2X7 receptor knockout mice in terms of aquaporin-5 reduction as wild type animals. Finally, P2X7 receptor function was examined by using the alveolar epithelial cell lines E10 and MLE-12 for stimulation experiments with bleomycin. The *in vitro* activation of P2X7 receptor was connected with an increase of aquaporin-5, whereas the inhibition of the receptor with oxidized ATP resulted in down regulation of aquaporin-5. The early loss of aquaporin-5 which can be found in different pulmonary fibrosis models does not implicate a specific pathogenetic role during fibrogenesis.

## Introduction

Alveolar epithelial type I (AT I) cells contribute 7% of total lung cells and cover over 95% of the alveolar surface. This thin epithelium allows the easy diffusion of gases and forms a barrier against the indiscriminate leakage of fluid into alveolar spaces. It also regulates the exchange of physiologically important solutes and water between circulating blood and the alveolar space. There is new evidence that AT I cells, in addition to alveolar epithelial type II (AT II) cells, contribute to active Na^+^ transport across the alveolar epithelium. They contain functional ion channels such as amiloride-sensitive epithelial Na^+^ channels (ENaC), cystic fibrosis transmembrane regulator (CFTR) and the water channel aquaporin-5 (AQP-5) [1]. Adenosine -5′- phosphate (ATP)-gated purinergic receptors (P2XRs), P2X7R and P2X4R were also identified in AT I cells [2]. These channels are extracellular ligand-gated ion channels. The ion channel is for mono- and divalent cations (Na^+^, K^+^, Ca^2+^) permeable and contains three functional domains: an extracellular domain that binds a native agonist, a transmembrane domain forming an ion channel pore, and an intracellular domain critical for regulation and crosstalk with various signaling pathways. Purinergic receptor signaling is involved in regulating many physiological and pathophysiological processes [3], [4].

The P2X7R, a member of the ionotropic P2X receptor family is selectively localized in AT I cells and plays probably an important part in mediating extracellular ATP signalling [5]. Recent animal studies have identified the importance of P2X7R and pannexin-1 complex in interleukin- (IL) 1β maturation, lung inflammation and development of pulmonary fibrosis [6].

Pulmonary fibrosis is characterized by inflammation and fibrosis of the interstitium and destruction of alveolar histoarchitecture leading finally to a fatal impairment of lung function. Fibroblasts and alveolar epithelial cells seem to be the principal players in this pathological process and interact in a paracrine fashion [7]. Pro-fibrotic cytokines such as transforming growth factor beta-1 (TGF-β1), platelet-derived growth factor (PDGF) and tumor necrosis factor alpha (TNF-α) are released causing fibroblast transformation, proliferation and accumulation of an extracellular matrix. Alveolar epithelial cells undergo i: increased epithelial cell death [8] and ii: several altered phenotypes e.g. AT II hyperplasia and epithelial mesenchymal transition (EMT) [9]. Injured alveolar epithelial cells in turn regulate fibroblasts and the activation of other lung cells. Surprisingly, knockout animals with deletion of AT I cell-specific antigens exhibit several pulmonary defects [10], [11]. Data about the pulmonary phenotype of P2X7R-deficient mice are missing. Recently, it was shown that bleomycin (BLM)-treated P2X7R-deficient mice have less neutrophil airway influx and inflammatory cytokine production with reduced pulmonary fibrosis in terms of lung collagen and matrix-remodeling proteins [6]. Water channels (aquaporins (AQPs)) are a family of water-specific membrane channel proteins found in cellular membranes. Four channels (AQP-1, AQP-3, AQP-4 and AQP-5) are expressed in the respiratory tract, with predominantly non-overlapping cellular and subcellular distributions [12] (with original references cited therein). The main AQPs in peripheral lung are AQP-5 in AT I cells and AQP-1 in endothelial cells. AQP-5, cloned originally from salivary gland [13], is an aquaporin with yet unknown molecular function in the lung. Under pathological circumstances increased or decreased levels of AQP-5 protein were found. It was shown that BLM-induced pulmonary inflammation in rats is associated with an increase of the expression of AQP-5 [14]. Gabazza et al. (2004) [15] demonstrated that lung fibrosis, the end-stage of a chronic process in the lung is associated with decreased protein and mRNA expression of AQP-5.

Previously it was demonstrated that the AQP-4 expression in brain is regulated by P2X7R activation *in vitro* [16]. They have shown that an activation of P2X7R by short-term 2′(3′)-O-(4-Benzoylbenzoyl)-ATP (BzATP) treatment significantly decreased AQP-4 protein expression, which was inhibited by the pretreatment of P2X7R antagonist oxidized ATP (oxATP). This could presumably involve the changes of intracellular Ca^2+^ and Protein kinase C (PKC)-*β*1 activity [17], [18], [19], which are known to decrease the osmotic water permeability of AQP-4 [20] or AQP-4 protein expression [21].

Our own *in vitro* experiments have demonstrated that BLM-treated alveolar epithelial cells show an increase in the expression of P2X7R, calmodulin (CaM) and PKC-*β*1. From this we predict that increased Ca^2+^ concentration stimulates PKC-*β*1, whereas the prerequisite for activating PKC-*β*1 after P2X7R increase remained to be determined [22].

As far as we know, a direct functional relationship between AQP-5 and P2X7R has not been established. Since AQP-5 and P2X7R are known to be present in AT I cells, we examined whether P2X7R knockout influences its pulmonary phenotype, particularly the AQP-5 expression. Further, we were interested in the involvement of AQP-5 in early processes of BLM-induced lung injury in P2X7R knockouts using precision cut lung slices and the immortal lung cell lines E10 and MLE-12.

## Materials and Methods

### Ethics Statement

All animal experiments were approved by the ethics committee of the Dresden University of Technology and the license for removal of organs was provided by the Landesdirektion Dresden (file no. 24-9168.24-1/2007-26; file no. 24-9168.24-1/2010-11).

### Mice

Wild type (C57BL/6) and *P2rx7* ^−/−^ mice (B6.129P2-*P2rx7^tm1Gab^/J*) were obtained from Pfizer (New York, NY, USA) [23]. Our animals were housed at Animal Care Facility at the Medical Faculty “Carl Gustav Carus” of Dresden University of Technology. The mice had always free costant access to standard chow and water. All performed procedures were in accordance with the Technical University Dresden Animal Care and Use Committee Guidelines. For our experiments we examined lung tissues from male and female mice at different ages: Younger mice were aged from 2 to 3 months while older mice were aged from 11 to 12 months. Mice were anesthetized with intraperitoneal lethal dose of 5% (w/v) thiopental (Thiopental 0.5 g, Inresa, Freiburg, Germany) or killed by cervical fracture, followed by lung removal.

### Cell Lines and Cell Culture

E10 cells (reported by Herzog et al. (1996) [24]) were kindly provided by M. Williams (Pulmonary Center, Boston University School of Medicine, Boston, MA, USA). For cultivation DMEM/Ham’s F12 medium was purchased from Gibco (Life Technologies, Carlsbad, CA, USA), which was supplemented with 5% (v/v) fetal bovine serum (PAN Biotech GmbH, Aidenbach, Germany) and 2.5 mM L-glutamine (Merck Millipore, Billerica, MA, USA). E10 cells were grown at 37°C in a 5% CO_2_ atmosphere up to a confluence of approximately 60–80%. They were seeded at a density of 3×10^4^ cells/ml and passaged three times per week (up to 25 passages). The mouse lung epithelial cell line MLE-12 [25] was cultured in complete HITES medium [26] containing: DMEM/Ham’s F12 (1∶1) (Life Technologies) supplemented with 4.5 mM L-glutamine (Merck Millipore), 2% (v/v) fetal bovine serum (Merck Millipore), 10 mM HEPES (Life Technologies), 0.005 mg/ml insulin, 0.01 mg/ml transferin, 30 nM sodium selenite, 10 nM hydrocortisone, 10 nM beta-estradiol (Sigma-Aldrich, St. Louis, MO, USA) and 1% (v/v) penicillin/streptomycin (Merck Millipore). Cells were cultured at 37°C in a 5% CO_2_ atmosphere up to 60–80% confluence and were seeded at a density of 6×10^4^ cells/ml. Cells were passaged continously two times a week to a maximum of 30 passages. For BLM treatment medium was completed by 100 mU/ml BLM (Cell Pharm GmbH, Bad Vilbel, Germany). Further we used oxATP (100 µM) from Sigma-Aldrich.

### Western Blot Analysis

Total protein concentrations of cell lysates were determined by performing amidoschwarz protein assay previously described by Dieckmann-Schuppert and Schnittler (1997) [27] whereas for murine tissue homogenization Precellys 24 Homogeniser (PEQLAB, Erlangen, Germany) and lysis buffer including 0.02 M Tris, pH 8.5; 0.125 M NaCl; 1% (v/v) Triton X-100 and protease inhibitor Complete Tablets, EDTA-free (Roche, Basel, Switzerland) were used. Pierce BCA Protein Assay Kit (ThermoFisher Scientific, Waltham, MA, USA) was used for measurement of protein concentration from lung tissue. For blotting 10–50 µg of total protein per sample were transferred into 6x SDS sample buffer (300 mM Tris-HCl, pH 6.8; 100 mM Dithiothreitol; 0.1% bromophenol blue; 30% (w/v) glycerol; 10% (w/v) SDS), boiled for 5 min at 95°C, loaded on a 12% SDS-polyacrylamide gel and blotted as accurately described by Linge et al. (2007) [28]. Proteins were transferred onto a 0.45 µm PVDF Immobilon-P Membrane (Merck Millipore, Billerica, MA, USA) and blocked in TBST buffer (17 mM Tris, ph 7.4; 2.7 mM KCl; 137 mM NaCl; 0.2% (v/v) Tween 20) including 2% (w/v) casein for 1 hour at room temperature (RT). Incubation with primary antibodies were done overnight at 4°C followed by 1 h lasting incubation with the secondary antibody at RT. Primary antibodies are listed in Table 1. The following secondary antibodies were used: donkey anti-rabbit IgG, HRP-linked F(ab)2 fragment (GE Healthcare, Chalfont St Giles, Buckinghamshire, United Kingdom), horse anti-mouse IgG, HRP-linked antibody (Cell Signaling, Danvers, MA, USA), and goat anti-Syrian hamster IgG-HRP (Santa Cruz, Dallas, TX, USA). Chemiluminescent signal was detected by following manufacture’s guidelines of Immobilon Western Chemiluminescent HRP Substrate (Merck Millipore) and by the use of Image Reader LAS-3000 (Fujifilm, Tokio, Japan). Quantification was done with ImageJ 1.43 u free software (Wayne Rasband, National Institutes of Health, Bethesda, MD, USA) and each lane was normalized to corresponding γ-Tubulin (γ-Tub) (Table 1).

**Table 1:** Primary Antibodies used for Western Blot (WB) and Immunohistochemistry (IHC).

Antibody	Dilution used for		Host	Type	Supplier
	WB	IHC			
anti-Aquaporin 5	1∶250	1∶200	Rabbit	Polyclonal	(Sigma-Aldrich, St. Louis, MO, USA)
anti-Caspase-3 (Asp175)	–	1∶50	Rabbit	Polyclonal	Cell Signaling (Danvers, MA, USA)
anti-Caveolin 1 clone 2297	1∶1000	–	Mouse	Monoclonal	BD Biosciences (Franklin Lakes, NJ, USA)
anti-Caveolin 1 (D46G3)	–	1∶1600	Rabbit	Monoclonal	Cell Signaling (Danvers, MA, USA)
anti-Connexin 43	1∶500	1∶2000	Rabbit	Polyclonal	(Sigma-Aldrich, St. Louis, MO, USA)
anti-P2X7	1∶500	–	Rabbit	Polyclonal	Alomone Labs (Jerusalem, Israel)
anti-Podoplanin (T1α) (8.1.1)	1∶1000	–	Hamster	Monoclonal	Santa Cruz (Dallas, TX, USA)
anti-Podoplanin (T1α)	–	1∶100	Hamster	Monoclonal	Kindly provided by M. Williams (Boston, MA, USA)
anti-γ-Tubulin clone GTU-88	1∶1000	–	Mouse	Monoclonal	(Sigma-Aldrich, St. Louis, MO, USA)
anti-CD44v10 clone k962	–	1∶1600	Rat	Monoclonal	Kindly provided by Dr. Ursula Guenthert (Basel, Switzerland)

### Conventional Histology

After organ removal, mouse tissue was fixed in 4% (v/v) buffered formalin at RT overnight, washed in PBS, dehydrated by incubating 1×20 min in 40% (v/v) ethanol (EtOH), overnight in 70% (v/v) EtOH, 2×20 min in 96% (v/v) EtOH, 3×20 min in 100% (v/v) EtOH, 2×20 min in xylene by following paraffin embedding at 60°C for 2 h. Sections of 5 µm were cut with a Jung Autocut 2055 microtome (Leica Microsystems, Wetzlar, Germany), mounted on SuperFrost® Plus slides (Langenbrück, Emmendingen, Germany) and dried overnight at 37°C. Next dewaxing was done by incubating the slides 3×5 min with xylene, followed by hydration 3×2 min in 100% (v/v) EtOH, 2×2 min in 96% (v/v) EtOH, 1×2 min in 70% (v/v) EtOH, 1×2 min in 40% (v/v) EtOH and 1×2 min in destilled water. Sirius Red staining of collagen and reticulin fibers was done as previously described by Kasper et al. (2004) [29] with a Sirius Red/fast green solution (0.1 g fast green and 0.1 g Sirius Red in 100 ml saturated picric acid) for 30 min at RT, followed by washing 1x with distilled water, dehydration (rapidly immersing slides in 96% (v/v) and 100% (v/v) EtOH 10x each), clearing 3×2 min in xylene and embedding in DePeX (Serva, Heidelberg, Germany).

### Immunohistochemistry

Embedding in paraffin, sectioning and dehydration were performed as already described above in 2.5. According to Kasper and Fehrenbach (2000) [30] by following the manufacturer’s instructions of Vectastain Elite ABC Kit (Vector Laboratories, Burlingame, CA, USA) slides were microwave irradiated 2×5 min at 1000 W in 0.01 M sodium citrate buffer (ph 6.0) followed by cooling down for 15 min. Slides were 2× rinsed with destilled water and treated with 0.3% hydrogen peroxide blocking solution for 30 min at RT. Afterwards slides were washed 2×5 min with PBS and blocked in 1.5% (v/v) goat serum for 20 min at RT. Subsequently the primary antibody was diluted in PBS and incubated overnight at 4°C. Used antibodies are shown in Table 1. Next day slides were washed 2×5 min in PBS and incubated with secondary biotinylated antibody for 45 min at RT. After washing 2×5 min in PBS, slides were incubated for 45 min at Vectastatin ABC Reagent at RT, followed by 2×5 min of washing in PBS. Afterwards sections were stained with 3,3′-Diaminobenzidine (DAB) for 5 min and washed with destilled water for 2×5 min. The following counterstaining was done with hematoxylin for approximately 20 sec. For embedding slides were washed in tap water for 10 min, dehydrated (1×2 min in 40% (v/v) EtOH; 1×2 min in 70% (v/v) EtOH; 2×2 min in 96% (v/v) EtOH; 3×2 min in 100% (v/v) EtOH; 3×2 min in xylene) and covered in DePeX (Serva, Heidelberg, Germany). For negative controls, PBS or non-immune serum was used instead of the primary antibody.

### RNA Isolation and Real-time Reverse Transcription PCR (Real-time RT PCR)

Isolation of total RNA was performed by using RNeasy Mini Kit (Qiagen, Hilden, Germany) according to the supplier’s instructions. RNA concentrations were determined with NanoPhotometer (Implen, Munich, Germany). For digesting DNA DNase I (ThermoFisher Scientific, Waltham, MA, USA) was applied. Synthesis of cDNA was done with Revert Aid H Minus First Strand Synthesis Kit (ThermoFisher Scientific) from 1–5 µg of total RNA using oligo-(dT)_18_ primers. Using CFX96 Real-Time PCR Detection System (Bio-Rad, Herkules, USA) and Maxima SYBR Green/Fluorescein qPCR Master Mix (ThermoFisher Scientific) for running PCR, the following conditions for all primer sets were used: initial denaturation 8 min at 95°C, followed by 40 amplification cycles (95°C for 20 sec; 58°C for 45 sec; 72°C for 20 sec) with a final extension step at 72°C for 2 min. Primers applied for real-time RT PCR are listed in Table 2. Identity of PCR products was proven by melting point analysis and agarose gel electrophoresis. Relative quantification of gene expression was done with the ΔΔCT method using CFX Manager (Bio-Rad) with *Polr2a* and *Rpl32* as housekeeping genes.

**Table 2:** Primers used for quantitative real-time RT PCR.

Gene	NCBI no.	Sequence	Product length
AQP5	NM_009701.4	5′-GCG CTC AGC AAC AAC ACA AC-3′	327 bp
		5′-CGA GGA GGG GAA AAG CAA GT-3′	
Cav-1	NM_001243064.1	5′-CGG GAA CAG GGC AAC ATC TA-3′	331 bp
		5′-CCC AGA TGT GCA GGA AGG AG-3′	
Cx43	NM_010288.3	5′-TGG GAT TGA AGA ACA CGG CAA G-3′	442 bp
		5′-AGG AGG AGA CAT AGG TGA GAG TGG-3′	
POLR2A	NM_009089.2	5′-TCG AGC AGA TCA GCA AGG TG-3′	213 bp
		5′-CAA TGC CCA GTA CCG TGA AG-3′	
RPL32	NM_172086.2	5′-GCA CCA GTC AGA CCG ATA TGT G-3′	78 bp
		5′-CTT CTC CGC ACC CTG TTG TC-3′	
T1α	NM_010329.2	5′-GGC GAG AAC CTT CCA GAA ATC-3′	145 bp
		5′-GGG ATG AAA CGC AGA CAA CAG-3′	

### Precision-cut Lung Slices and Tissue Culture

Following the preparation procedure of precision-cut lung slices (PCLS) described by Held et al. (1999) [31] tissue preparation was done with several modifications. Wild type and *P2rx7^−/−^* mice up to an age of 3 months were anesthetized with an overdose of 5% (w/v) thiopental (Thiopental 0.5 g, Inresa, Freiburg, Germany) including 600 international units (IU) heparin (Ratiopharm, Ulm, Germany) and exsanguinated by cutting the left renal artery. The chest wall was opened and the lungs were perfused with 10 ml of room tempered PBS through the right ventricle. Subsequently the trachea was cannulated with a 20-gauge catheter (Saf-T-Intima with Y adapter, BD Biosciences, Franklin Lakes, NJ, USA) and the lungs were filled with 1% (w/v) low-melting agarose (Sigma-Aldrich, St. Louis, MO, USA) solved in PBS at 37°C *in situ*. For agarose solification the mouse cadaver was cooled with ice for 15 min at 4°C. Next, solidified lungs were removed and separated into the different lobes. Each lobe was embedded in 3% (w/v) prewarmed agarose at 42°C. For gelling, agarose was cooled with ice at 4°C for 15 min. Afterwards each lobe was cut with Krumdieck tissue slicer (Alabama Research and Development, Munford, AL, USA) into 500 µm thick slices using DMEM/F12 medium with 15 mM Hepes and 2.5 mM L-glutamine (Life Technologies, Carlsbad, CA, USA) cooled down to 4°C. Then tissue slices were transferred into cell culture plates containing above-mentioned medium including 1% (v/v) pen/strep (Merck Millipore) and 0.5% (v/v) FCS (PAN Biotech GmbH) at 37°C. During the following 3 h medium was changed every 30 min. For add-on tissue culture two lung slices each were transferred on one Teflon mesh insert (Roller Insert Type A, Vitron Inc, Tucson, AZ, USA) and placed into a 20 ml glass vial (Scintillation Vial, KG-33 Borosilicate Glass, VWR International, Radnor, PA, USA) containing 1.7 ml of medium. For BLM treatment medium was completed by 200 mU/ml BLM (Cell Pharm GmbH, Bad Vilbel, Germany). Glasses were closed with Filter Easy Caps 25 cm^2^ (ThermoFisher Scientific, Waltham, MA, USA). The vials were put in a roller system (Stuart Rotator SB3, Bibby Scientific, Staffordshire, United Kingdom) rotating at 5 rpm at 37°C and 5% CO_2_. Every 24 h medium was changed. After 72 h of incubation the lung slices were homogenized for Western Blotting or embedded in paraffin for immunostaining as explained above.

### Statistical Analysis

The statistical computation was done upon the recommendation of Dresden Institute for Medical Informatics and Biometry (Dresden, Germany). Statistical analysis was performed pretesting for a Gaussian distribution with Shapiro-Wilk-Test followed by an unpaired and two-tailed student t test. In case of declining normal distribution significance was tested by Wilcoxon-Mann-Whitney-Test (U-Test). Results were usually presented as Box-Whiskers-Plots including the minimum, the 25th percentile, the median, the 75th percentile and the maximum of all values. For cell experiments including bleomycin treatment results were presented as mean ± standard error of mean (SEM) in relation to the control. The whole statistical analysis was done with GraphPad Prism 5.03 software (GraphPad Software, San Diego, CA, USA). Significance was accepted when the p value was below 0.05 (indicated by *). A minimum of three independent experiments was done.

## Results

### P2rx7^−/−^ Knockout Animals Show Reduced Aqp5 mRNA Expression and AQP-5 Protein Levels

**Figure 1:**
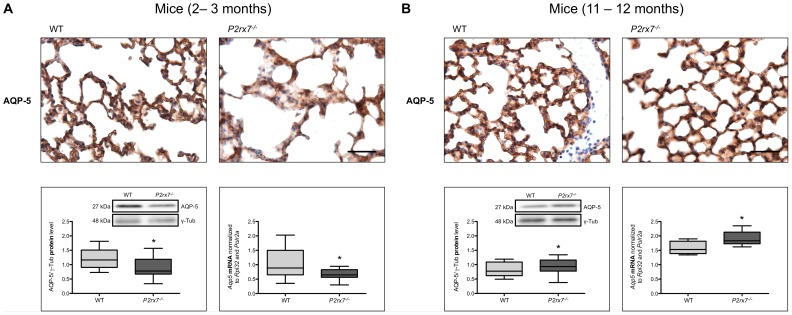
Protein content and expression of AQP-5 in *P2rx7^−/−^* mice lungs. Immunohistochemical staining of AQP-5, *Aqp5* mRNA expression and analysis of AQP-5 protein content in lungs from wild type and *P2rx7^−/−^* mice aged (A) 2–3 months and (B) 11–12 months. Scale bar correlates to 50 µm in immunoperoxidase demonstrations. Quantitative real-time RT PCR was done by using *Rpl32* and *Polr2a* as housekeeping genes. Results are shown median with whiskers extending to the minimum and maximum (n_(2–3 months)_ = 18, n_(11–12 months)_ = 9). Equal protein amounts were used in SDS-PAGE and analyzed by western blot with the antibody against AQP-5. Relative protein levels AQP-5/γ-Tub are shown median with whiskers extending to the minimum and maximum (n_(2–3 months)_ = 15, n_(11–12 months)_ = 15) and one representative blot is pictured (* p<0.05).

In different lung fibrosis models, a loss of AQP-5 from alveolar epithelial cells has been described, thus indicating AQP-5 as an early injury marker in lung [32], [33]. The alveolar epithelium plays a central role in gas exchange and fluid transport, and is therefore critical for normal lung function. Immunohistochemical assessment of lungs from wild type and *P2rx7* ^−/−^ mice *in situ* revealed significant differences in AQP-5 immunoreactivity. Decreased levels of AQP-5 protein expression were detected in *P2rx7* ^−/−^ mice compared to wild type in 2–3 month old animals (Figure 1A). In parallel, western blot analysis revealed decreased protein content of AQP-5 in *P2rx7^−/−^* mice (Figure 1A). Quantitative real-time RT PCR analysis showed less abundant content of *Aqp5* mRNA in the homogenates of lungs from *P2rx7^−/−^* mice compared to the control group (Figure 1A).

Quantitative real-time RT PCR and western blot analysis of lungs from 11–12 month old wild type and *P2rx7* ^−/−^ mice have shown a slightly increase of AQP-5 in the *P2rx7* ^−/−^ mice (Figure 1B). Immunohistochemical assessment of lungs from these animals *in situ* showed no increase in AQP-5 immunoreactivity in the *P2rx7* ^−/−^ mice (Figure 1B).

### Analysis of Fibrosis-relevant Proteins in P2rx7^−/−^ Animals Over a Period of 12 Months

It was shown that chronic lung injury and lung fibrosis is associated with decreased protein and mRNA expression of AQP-5 in the lung [15], [33]. Therefore, we have analyzed the *P2rx7^−/−^* mice over a period of 12 months to determine whether the absence of the P2X7R together with reduced AQP5 protein content is spontaneously leading to the development of fibrosis-like lesions.

We have examined the *P2rx7^−/−^* mice on alterations similar to changes observed in early stages of lung fibrogenesis: (1) extracellular matrix accumulation, (2) alveolar epithelial changes, particularly AT I cell injury with loss of AT I cell specific antigens, and (3) increased apoptosis of pulmonary cells. We included in this study 2–3 and 11–12 month old animals.

**Figure 2:**
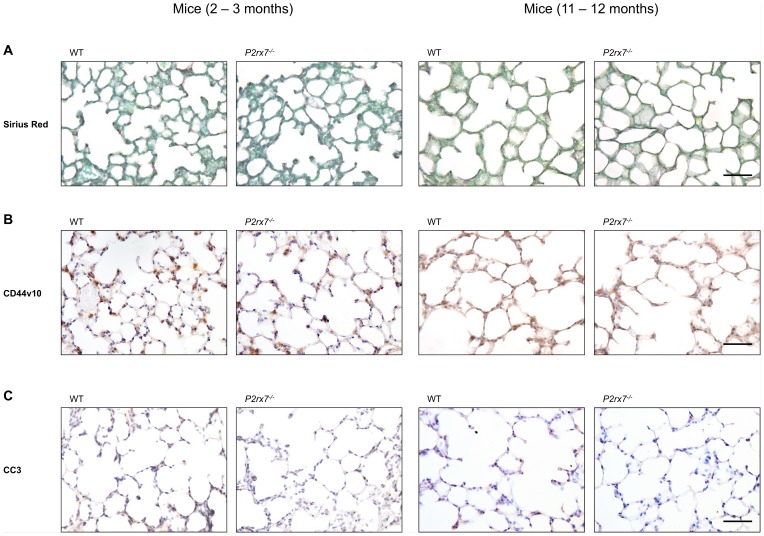
Sirius Red staining and immunohistochemical staining of CD44v10 and CC3 in *P2rx7^−/−^* mice lungs. Immunoperoxidase demonstration of Sirius Red (A), CD44v10 (B) and CC3 (C) in lungs from two cohorts of wild type and *P2rx7^−/−^* mice (younger age bracket 2–3 months; older age bracket 11–12 months). Scale bar 50 µm.

A hallmark of fibrosis is the presence of fibroblastic foci with differentiated fibroblasts which show myofibroblast phenotypes and which secrete in abundance extracellular matrix proteins [34]. Using Sirius Red staining, we found no differences of total collagen in *P2rx7^−/−^* mice in comparison to the wild type mice regardless of age (Figure 2A). Previously we have shown that immunohistochemical assessments of lungs from wild type and *P2rx7^−/−^* mice revealed no changes in the protein content for collagen type I [22]. For determination of the expression of collagen1a1 and collagen1a2, total RNA from cell homogenates of wild type and *P2rx7^−/−^* mice were prepared and subjected to quantitative real-time RT PCR. The collagen1a1 and collagen1a2 expression in the homogenates of lungs from 14 wild type and 17 *P2rx7^−/−^* mice showed no differences in the expression of mRNA (data not shown).

In the normal lung, AT II cells express CD44 epithelial isoforms (CD44v1-10) predominantly at the basolateral site indicating a function as adhesion protein mediating contact to the underlying matrix [35]. CD44 is further involved in uptake and degradation of hyaluronate in lung and lymphoid tissue [36], [37]. Advanced stages of disease could show that alveolar epithelial cells in fibrotic foci were either devoid of CD44 epithelial isoforms or overexpressed at places with epithelial hyperplasia [35]. Immunocytochemical detection of CD44v10 expression in paraffin sections of lungs from *P2rx7^−/−^* and wild type mice revealed no differences in the CD44v10 expression pattern between *P2rx7^−/−^* and wild type mice regardless of age (Figure 2B), thus indicating normal AT I and AT II differentiation.

Immunohistochemically detectable differences of apoptosis in lung parenchymal cells were analyzed by detection of active caspase-3. The level of apoptosis in the *P2rx7^−/−^* mice was comparable with the wild type mice (Figure 2C).

**Figure 3:**
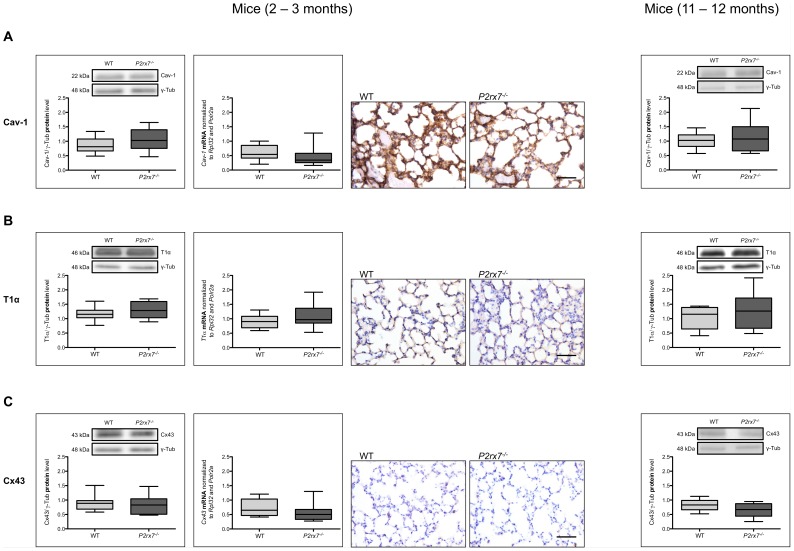
Protein content and expression of Cav-1, T1α and Cx43 in *P2rx7^−/−^* mice lungs. mRNA expression, analysis of corresponding protein content and immunoperoxidase demonstration of Cav-1 (A), T1α (B) and Cx43 (C) in lungs from wild type and *P2rx7^−/−^* mice aged 2–3 months compared to the particular protein content in wild type and *P2rx7^−/−^* mice aged 11–12 months. Quantitative real-time RT PCR was done by using *Rpl32* and *Polr2a* as housekeeping genes. Results are shown median with whiskers extending to the minimum and maximum (n = 18). Equal protein amounts were used in SDS-PAGE and analyzed by western blot with antibodies against the particular protein. Protein levels are normalized to γ-Tub and are shown median with whiskers extending to the minimum and maximum (n_(2–3 months)_ = 15, n_(11–12 months)_ = 10) and one representative blot is pictured. Scale bar correlates to 50 µm in immunoperoxidase demonstrations.

Caveolin-1 (Cav-1) is an integral membrane protein of AT I and other lung cells. Its relevance in fibrotic diseases has already been recognized in earlier studies [38], [39]. The *Cav-1* expression in the homogenates of lungs from 2–3 month old *P2rx7^−/−^* mice showed no differences in comparison to wild type mice. Western blot analysis of lung homogenates showed also no differences in the Cav-1 content in the *P2rx7^−/−^* mice both in 2–3 month as well as in 11–12 month old animals (Figure 3A). Immunohistochemical assessment of lungs from wild type and *P2rx7^−/−^* mice showed no differences in the immunoreactivity for T1α, an AT I cell specific protein and early marker of lung injury in fibrosis [40]. Western blot analysis and quantitative real-time RT PCR of lung homogenates demonstrated also no differences in *P2rx7^−/−^* and wild type mice regardless of age (Figure 3B).

Connexins, the elementary units of intercellular gap junctions, are known to be expressed in AT I and II cells and hemichannel activity shows some functional relationship to P2X7R activity [41].

Enhanced immunoreactivity for connexin 43 (Cx43) was detected in epithelial cells at earliest stage during the development of radiation-induced pulmonary fibrosis [30]. Immunohistochemical assessments and western blot analysis from 2–3 month old wild type and *P2rx7^−/−^* mice showed no significant differences in the amount of Cx43 (Figure 3C). Quantitative real-time RT PCR analysis from 2–3 month old animals showed no significant differences in the content of *Cx43* mRNA in the homogenates of lungs from *P2rx7^−/−^* mice compared to the control group (Figure 3C). Western blot analysis from 11–12 month old animals showed also no significant differences in the content of Cx43.

Altogether, all proteins with putative properties as early indicators of fibrosis in alveolar epithelial cells showed no alterations in *P2rx7^−/−^* mice during the course of age.

### Immunohistochemistry of PCLS from Lungs of Wild Type and P2rx7^−/−^ Mice Revealed Decreased Expression of AQP-5 in AT I After BLM Treatment

**Figure 4:**
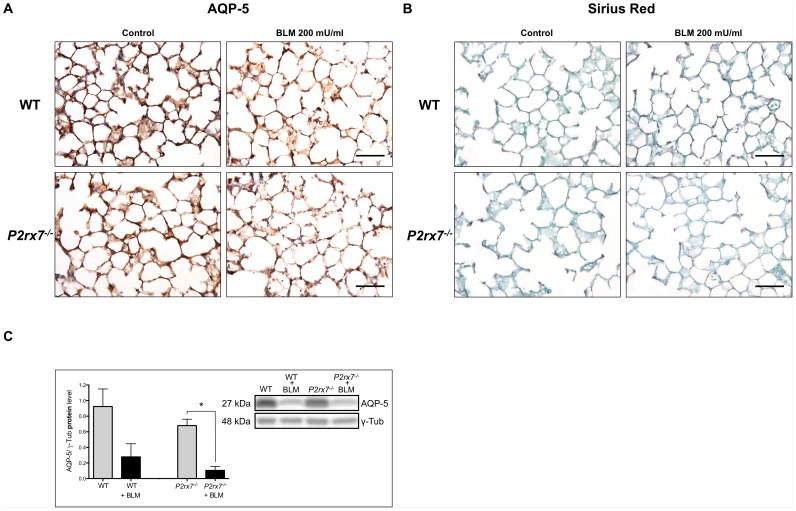
Collagen and AQP-5 protein content in wild type mice compared to *P2rx7^−/−^* knockout mice after BLM treatment. Immunoperoxidase demonstration of AQP-5 (A) and Sirius Red staining (B) of wild type and *P2rx7^−/−^* mice lungs after BLM treatment with 200 mU/ml for 72 h in PCLS. Untreated lung slices after 72 h were used as controls. Scale bar 50 µm. For analysis of AQP-5 protein content, equal protein amounts were used in SDS-PAGE and analyzed by western blot with the antibody against the AQP-5 (C). BLM treated and untreated PCLS from wild type and *P2rx7^−/−^* mice lungs were compared statistically. Relative protein levels AQP-5/γ-Tub are shown mean ± SEM (n = 4) and one representative blot is pictured (* p<0.05).

Figure 4 illustrates the immunohistochemical assessment of paraffin embedded PCLS prepared from 1–3 month old wild type and *P2rx7^−/−^* mice before and after BLM exposure. After 3 days of BLM treatment a decrease in AQP-5 protein content was detected in PCLS of lungs of the wild type mice in comparison to the untreated wild type mice (Figure 4A, C). Interestingly, the *P2rx7^−/−^* mice treated with BLM showed a stronger reduction in the AQP-5 protein content in comparison to the untreated *P2rx7^−/−^* mice. To analyze the level of early fibrosis, we assessed also collagen by using conventional Sirius Red staining (Figure 4B). There were no signs of overproduction of collagen after BLM exposure of PCLS from wild type and knockout animals under the described conditions.

### The AQP-5 Expression is P2X7R-dependent

**Figure 5:**
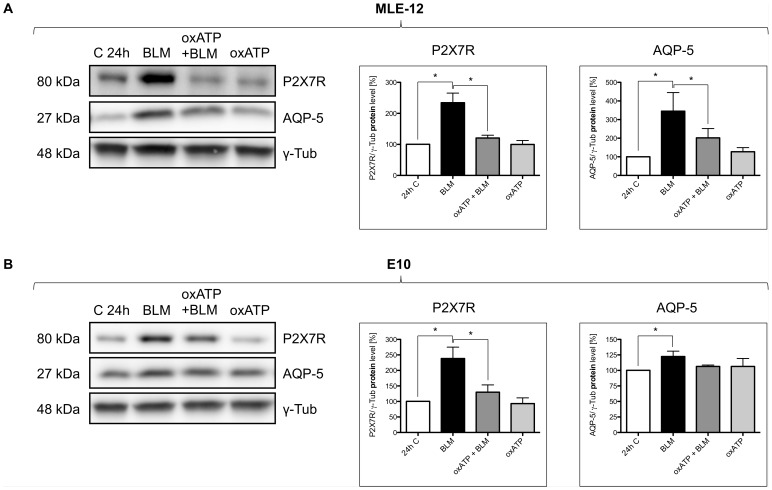
Dependency of AQP-5 on P2X7R expression. Analysis of protein content in MLE-12 cells (A) and E10 cells (B) after BLM treatment: Cells were treated with 100 mU/ml BLM, 100 µM oxATP with BLM (100 mU/ml) and 100 µM oxATP for 24 h. oxATP was added 2 h prior to BLM treatment. Untreated cells were used as control. For SDS-Page equal protein amounts of cell lysates were used and analyzed by western blot with antibodies against P2X7R and AQP-5. Protein levels are normalized to γ-Tub and are shown mean ± SEM (n = 6) in relation to control. One representative blot is pictured (* p<0.05).

Finally we analyzed whether the stimulation of P2X7R in E10 and MLE-12 cells with BLM induces changes in the AQP-5 protein expression. An increase of the AQP-5 expression was observed after 24 h in both cell lines exposed to BLM (Figure 5A, B). To test the hypothesis, that the P2X7R is responsible for the increased AQP-5 expression after BLM treatment, we checked the AQP-5 expression after inhibition of the P2X7R by oxATP after 24 h. The resulting western blots demonstrated a decreased amount of AQP-5 after oxATP treatment in comparison to BLM treated cells (Figure 5 A, B).

**Figure 6:**
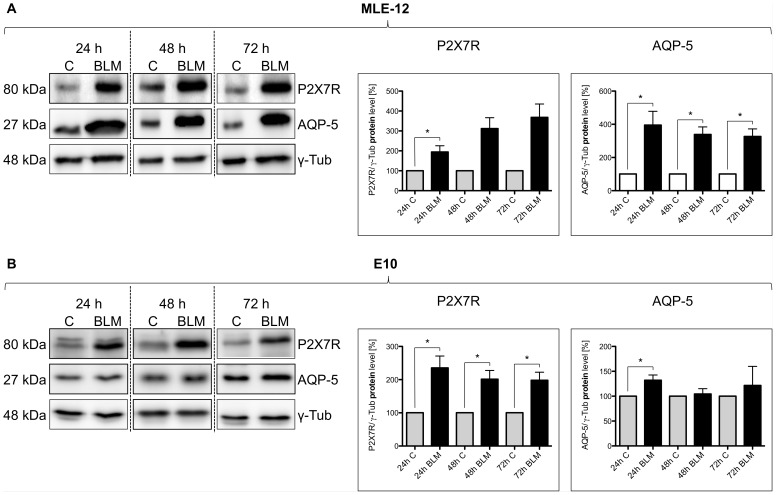
AQP-5 and P2X7R expression over the course of time. Analysis of protein content in MLE-12 cells (A) and E10 cells (B) after BLM treatment: Cells were treated with 100 mU/ml BLM for 24 h, 48 h and 72 h. Untreated cells after 24 h, 48 h and 72 h were used as control. For SDS-Page equal protein amounts of cell lysates were used and analyzed by western blot with antibodies against P2X7R and AQP-5. Protein levels are normalized to γ-Tub and are shown mean ± SEM (n = 3) in relation to control. One representative blot is pictured (* p<0.05).

To study the time-course of AQP-5 and P2X7R protein expression levels, E10 and MLE-12 cells were lysed after 24, 48 and 72 hours of BLM treatment (100 mU/ml) and subjected to Western blot analysis. P2X7R and AQP-5 protein expression are significant increased after 24 h exposure to BLM in both cell lines. Also after 48 and 72 hours the protein content of AQP-5 and P2X7R is increased (Figure 6 A, B).

The time course of AQP-5 of PCLS from wild type mice treated with BLM (200 mU/ml) have shown, however, an increase in AQP-5 after 24 h and a decrease of AQP-5 after 48 and 72 h exposure to BLM (data not shown). A direct comparison of both experimental settings is not possible, since we needed to use lower concentration of BLM in the cell culture model, to prevent the induction of apoptosis in E10 and MLE-12 cells.

## Discussion

Our results in the present study indicate that *P2rx7^−/−^* mice during the course of age did not develop any signs of pulmonary fibrosis or any other signs of lung injury. We could not find any enhancement or alteration in the expression of proteins, which can be regarded as early indicators of fibrosis such as collagen accumulation, loss of T1α [40] increase in CD44v10 or Cx43 [42], [43], [44]. Moreover, Riteau et al. (2010) [6] showed that the development of a fibrosis is reduced in absence of the P2X7R by demonstrating that the amount of collagen was strongly reduced in BLM treated *P2rx7^−/−^* mice.

In the present study, we have shown that as a prominent feature AQP-5 is downregulated in the 2–3 month old *P2rx7^−/−^* animals. This reduction was reversible, moreover AQP-5 expression increased strongly in older animals, where also no fibrotic lesions in the lung parenchyma were detectable. From these findings we suggest that the early loss of AQP-5 described in different fibrosis models [15] is not necessarily connected with the development of this disease but somehow connected with the function of other proteins of AT I cells. In some animals deficient in AT I cell-specific antigens, the lung *Aqp5* mRNA is also decreased [10], [11]. For example the T1α knockout mice express about 50% of the normal levels of *Aqp5* mRNA [10]. It was previously shown that T1α is neither involved in water transport nor regulates AQP-type water channels [45].

As a second outcome of the present study, PCLS of wild type and *P2rx7^−/−^* mice treated with BLM showed a decrease in the AQP-5 protein content. Remarkably, PCLS of the *P2rx7^−/−^* mice treated with BLM showed a stronger decrease in the protein expression of AQP-5 compared to untreated *P2rx7^−/−^* mice. The reason for this decrease is not clear. Treatment of PCLS with bleomycin did also not result in the development of fibrosis-like changes in the lung.

The alveolar epithelium seems to play a central role in gas exchange and regulation of lung water content in response to acute and subacute lung injury. AT I cells exhibit the highest water permeability ever reported for a mammalian cell [46]. The AT I cells likely regulate alveolar fluid volume, composition and clearance. The bulk of water flux across this epithelium depends on the AT I cell specific membrane water channel AQP-5. The airspace-capillary water permeability in AQP-5 knockout mice has a 90% decrease, suggesting the importance of AQP-5 under normal conditions in the lung [1]. Gabazza et al. (2004) [15] demonstrated that chronic lung injury and lung fibrosis is associated with decreased expression of AQP-5 in the lung. In the present study, treatment of immortal lung cell lines with BLM resulted in an increase in AQP-5 protein content. This obvious discrepancy between the PCLS and cell culture data, decreased AQP-5 after BLM exposure on the one side and increased AQP-5 on the other side, tangents a general problem in the comparison of experiments at the organoid level (explant culture) with experiments at the single cell level. We cannot exclude additional indirect effects derived from the absence or presence of other lung cell types in the present experimental settings. Nevertheless, we further demonstrated in the present study that P2X7R activation in alveolar epithelial cells by BLM is implicated in the upregulation of AQP-5 protein expression *in vitro*. The signal transduction of the P2X7R is supported mainly by the depolarization of the cell membrane and by increasing the intracellular Ca^2+^ concentration [47]. CaM is a ubiquitous Ca^2+^ sensor in which the binding regulates the activity of various ion channels, and thereby the ion balance of the cell can be maintained [48], [49]. Previously we have shown that BLM treated alveolar epithelial cells show an increase in the expression of P2X7R and CaM [22].

CaM is a Ca^2+^-binding protein and a universal regulatory protein that communicates the presence of calcium to its molecular targets and correspondingly modulates their function. Many membrane proteins that act as transporters and channels, such as the cardiac and neuronal voltage-gated ion channels [50], [51], [52], [53], gap junctions [54], [55] and the aquaporin water channels [56], [57] are modulated by CaM in response to cytoplasmic Ca^2+^ fluctuations.

Lipid rafts are known as cholesterol- and glycolipid-enriched microdomains [58] and have been implicated in membrane sorting and trafficking, as well as receptor signalling [58], [59], [60]. As previously reported, cevimeline induces the translocation of AQP-5 with lipid rafts from the cytoplasm to the apical plasma membrane (APM) by Ca^2+^ signalling and the dissociation of AQP-5 from lipid rafts to non-rafts in the APM [61]. It is noteworthy that lipid rafts are implicated in the sorting of some membrane proteins to the APM domain of epithelial cells [62]. Studies using membrane cholesterol depleting reagents, for example statins, have supported the role of membrane rafts in regulation of AQP-2 trafficking [63]. Previously, we have shown that two populations of the P2X7R are detectable in the plasma membrane of E10 cells. A discrete population of P2X7 was associated with lipid rafts [64].

In summary, the results of the present study show, that an activation of P2X7R *in vitro* is connected with an increase of AQP-5. Otherwise, if P2X7R is inhibited, a down regulation of AQP-5 was observed. These data suggest that P2X7R effects AQP-5 activity directly or indirectly. It may be that CaM is directly involved in these processes. The activation of the P2X7R could lead to the translocation of AQP-5 in lipid rafts. Further studies will be necessary to clarify the relationship between P2X7R activation and a potential localization of AQP-5 in lipid rafts of AT I cells.
